# Symptomatic Giant Cavernous Haemangioma of the Liver: Is Enucleation a Safe Method?

**DOI:** 10.1155/1997/53453

**Published:** 1997

**Authors:** H. Demiryürek, Ö. Alabaz, D. Ağdemir, I. Sungur, E. U. Erkoçak, A. Akinoğlu, A. Alparslan, S. Źorludemir

**Affiliations:** 1 Faculty of Medicine Department of Surgery University of ukurova Adana Turkey; 2 Department of Pathology Adana Turkey

## Abstract

Twenty-three patients with symptomatic giant hemangioma of the liver were treated by surgery between 1979 and 1996 at the department of General Surgery, Faculty of Medicine, University of Çukurova. Twenty-three enucleations were performed in 21 patients, left lateral segmentectomy in one patient and enucleation plus left lobectomy in one patient. The tumors were enucleated along the interface between the hemangioma and normal liver tissue. The diameters of the tumors ranged from 5×5 to 25×15 cm. The mean blood loss for enucleations was 525 ml (range 500–1000 ml). There was no mortality and no postoperative bleeding. Three patients had postoperative complications. Enucleation is the best surgical technique for symptomatic giant hemangioma of the liver. It may be performed with no mortality, low morbidity and the preservation of all normal liver parenchyma.


*HPB Surgery*, 1997, Vol. 10, pp. 299–304
Reprints available directly from the publisher
Photocopying permitted by license only

© 1997 OPA (Overseas Publishers Association)
Amsterdam B.V. Published in The Netherlands
by Harwood Academic Publishers
Printed in India

### Symptomatic Giant Cavernous Haemangioma of the Liver: Is Enucleation a Safe Method?

### A Single Institution Report

H. DEMIRYÜREK[a], Ö. ALABAZ[a], D. AĞDEMIR[a], I. SUNGUR[a], E. U. ERKOÇAK[a], A. AKINOĞLU[a], A. ALPARSLAN[a] and S. ZORLUDEMIR[b]

[a]*Faculty of Medicine, Department of Surgery, University of Çukurova;* [b]*Department of Pathology, Adana, Turkey*

*(Received 1 August 1996; In final form 27 November 1996)*

**Twenty-three patients with symptomatic giant hemangioma of the liver were treated by surgery between 1979 and 1996 at the department of General Surgery, Faculty of Medicine, University of Çukurova. Twenty-three enucleations were performed in 21 patients, left lateral segmentectomy in one patient and enucleation plus left lobectomy in one patient. The tumors were enucleated along the interface between the hemangioma and normal liver tissue. The diameters of the tumors ranged from 5×5 to 25×15 cm. The mean blood loss for enucleations was 525 ml (range 500–1000 ml). There was no mortality and no postoperative bleeding. Three patients had postoperative complications. Enucleation is the best surgical technique for symptomatic giant hemangioma of the liver. It may be performed with no mortality, low morbidity and the preservation of all normal liver parenchyma.**

*Keywords:* Liver, hemangioma, enucleation

### INTRODUCTION

Hemangiomas are probably the most common of all liver tumors, and the most frequent is cavernous hemangioma of mesenchymal origin. The etiology of hemangioma is still a matter of speculation. They vary in size from a few millimeters to many centimeters. Small hemangiomas of the liver are usually symptomless, but giant hemangiomas may be symptomatic.

The natural history of hemangiomas and the indications for surgical or conservative treatment are controversial. Surgical excision is the only definitive treatment [1]. Enucleation of symptomatic giant cavernous hemangiomas of the liver is emerging as an alternative surgical technique. We report our personal experience with enucleation of liver hemangiomas over the last 16 years.

### MATERIAL AND METHODS

Between January 1979 and January 1996, 23 patients underwent operations for symptomatic

Correspondence to: Associate Prof. Haluk Demiryürek, University of Çukurova, Faculty of Medicine, Department of Surgery, 01330 Balcali/Adana Turkey.

giant hemangiomas of more than 5 centimeters in diameter. We excluded asymptomatic patients or hemangiomas less than 5 centimeters in diameters. There were 18 women and five men aged 30 to 62 years (Mean 46 . 7 ). The indications for surgery were severe right upper quadrant pain radiating to right shoulder in six patients, abdominal discomfort, nausea and vomiting in three patients, painfull mass felt on physical examination in ten patients, obstructive jaundice in two patients, and misdiagnosed hepatocellular carcinoma in two patients. Biochemical evaluation was normal in all but two patients who had obstructive jaundice. The lesions were visualised on radionuclide scans in 4 patients, by ultrasonography combined with computerized tomography in 19. The follow-up data was obtained by examining the hospital record and an attempt was made to contact them by telephone and letter.

### Operative Technique

Upper midline and subcostal incisions were used. The falciform and triangular ligaments were divided and the liver was mobilized completely. The Pringle maneuver with a soft clamp was used to achieve inflow control. It was performed to control bleeding from the enucleation site in the right or left liver in five patients, for centrally located hemangiomas in three patients and following resection procedures for controlling blood loss from the raw surface of the liver in two patients. The Pringle maneuver consisted of periods of 20 min. clamping of the hepatic pedicle followed by 5 min. periods of restoration of blood flow.

After incising the liver capsule, finger dissection was performed in the plane between the capsule of the hemangioma and the surrounding liver tissue. Enucleation was accomplished step by step ligating the vessels entering or leaving the tumor avoiding entry into either the liver parenchyma or into the tumor itself.

After removal of the tumor, the defect in the liver was packed with warm gauze swabs and small bleeding points were controlled by catgut sutures. When the defect formed a large cavity in the liver parenchyma and minimal oozing persisted inside the cavity despite catgut sutures, the omentum was mobilized and placed in the cavity to prevent formation of an infected hematoma and secondary infection of the cavity. A vacuum drain was used for small cavities, but if the cavity was large, a silastic tube drain were placed adjacent to the cavity. Drains were removed within two or three days.

### RESULTS

The hemangiomas were solitary in 20 patients and multiple in 3 patients. The tumor was located in the right lobe in 14 patients, in the left lobe in 6 patients and in both lobes in 3 patients.

The tumor dimensions were determined by US and/or CT, and during operation. Hemangiomas varied in size from 5×5 to 25×15 cm. Twenty-three enucleation were performed in 21 patients, left lateral segmentectomy in one patient and enucleation plus left lobectomy in one patient. In the two patients in whom resections were performed, the hemangiomas were deeply located in the liver parenchyma. Obstructive jaundice in two patients due to pressure of the tumor on the common hepatic duct was demonstrated by ultra sonography and computerized tomography. After removal of the tumor, the common bile duct was explored with operative cholangiography and it was found to be normal. These patients became non-jaundiced after operation.

The mean blood loss during enucleations were 525ml (range, from 500 to 1000 ml). There were no postoperative bleeds and there were no deaths. Three patients had postoperative complications. One patient developed pneumonia, one had a wound infection and one had subphrenic abcess caused by displacement of silastic tube. The subphrenic abcess was treated by percutaneous drainage. The postoperative biochemical evaluation was normal in all patients. The follow-up period ranged from 1 to 16 years (mean 7.4 years). Five patients were unavailable for follow-up. Sixteen of remaining eighteen patients were well and asymptomatic. Mild pain persisted in the epigastric area in two patients.

### DISCUSSION

Cavernous hemangiomas of the liver are found at autopsy in 2 to 7 % of the population [2]. They affect women predominantly and they are most frequently encountered in the third, fourth, and fifth decades of life [3]. The surgical management of giant cavernous hemangiomas was described as early as 1942 by Schumacker [4], and recently there has been renewed interest in these tumors because of the advent of noninvasive imaging techniques [5,6,7]. Gaint hemangioma of the liver have been variously defined as a lesion greater than 4,6, or 8 cm. in diameter [8,9,10]. Giant hemangiomas may become symptomatic by pressure on adjacent organs or the liver. The most common symptom is the pain and mass effect.

Which cavernous hemangioma should be treated by surgery? The answer must depend upon balancing the risks of operation against the natural history of untreated lesions [11]. From literature and from our experience, surgical treatment should be performed to relieve the symptoms of the hemangioma and to treat the complications of the hemangioma such as thrombocytopenia, abcess formation, jaundice, rupture, and pressure symptoms [12,13,14,15]. Operation for asymptomatic lesions cannot be routinly recommended even if they are large because the majority of these lesions do not increase in size [16] . Which operation should be performed in patients who merit surgery? Most authors reported that the surgical strategy was decided on the basis of the size and the location of the lesions. When deciding on surgical management, the operative morbidity and mortality must be considered. Most authors report major hepatic resection for large tumors and perform segmentectomy or enucleation only for smaller lesions [17,18,19,20]. Schwartz [21] concludes that most large tumors are best resected along anatomical planes i.e. a lobectomy or trisegmentectomy with inflow vascular occlusion. Resection of large lesions may be attended by significant blood loss, high postoperative complication and the lost normal functioning liver parenchyma [11,19,21]. Since the hemangioma is a benign tumor, a technique of removing it that will spare the parenchyma seems desirable. The enucleation technique accomplishes this and it can be performed safely because involutional changes occur in many lesions resulting in a firm fibrous capsule, presumably as a consequence of the compression of the liver tissue caused by the growing tumor. Microscopically the tumors are made up of large blood-filled cavernous sinues lined with endothelium and separated by fibrous septae of varying thickness. They are usually encapsulated by a rim of fibrous tissue and the border between hemangioma and normal liver is distinct. Histologic examination demonstrates this plane (Fig. 1). In this interface between hemangioma and normal liver tissue, the hemangioma can be effectively enucleated and the vessels entering or leaving the tumor can be ligated and divided one by one. Vascular inflow occlusion may be used intermittently. Enucleation was first described by Alper *et al.* in 1988 [22], and thereafter Baer *et al.* [23], Petri *et al.* [24] and Kuo *et al.* [25] reported their experiences. In our institution, we have been using this surgical technique since 1979. Pichlmayr [26] reported that the enucleation technique can be performed safely even with total vascular occlusion when the hemangioma is very large or at a dangerous anatomical location adjacent to the inferior vena cava or a major hepatic vein. Since the enucleation occurs along the relatively avascular cap-

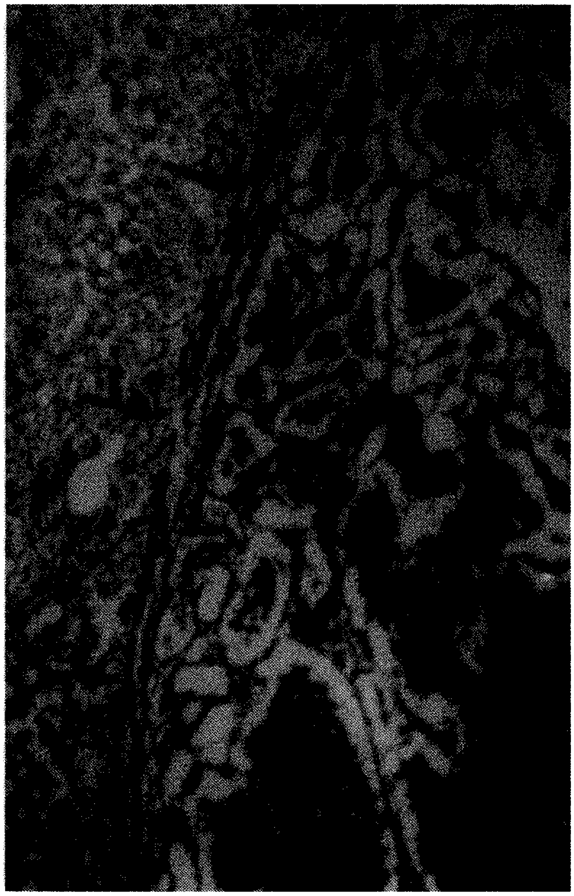

FIGURE 1 The technique of the enucleation was performed along the interface between and normal liver paranchyma that is distinguished by a capsule like fibrous septae (arrows) (H.E.×100).

sular plane, the amount of normal functioning parenchyma removed is minimal. So, the disturbance of liver function is usely insignificant. The residual cavity can be dealt with by meticulous dissection, good hemostasis combined with or without omentum placement in the cavity. In our experience with enucleation technique, there was no excessive blood loss and no mortality; the complication rate was rather low (15%). When the hemangioma is deeply located and has an infiltrative character into the liver parenchyma, the best approach for symptomatic giant hemangioma is the formal resection [27].

We think that symptomatic gaint hemangiomas are best treated by enucleation, no matter what size.

### *References*

[1] Schwartz, S. I. and Hemangioma (1995). In: Cameron, J. L., ed. (1995). *Current Surgical theraphy*, Mosby- Year Book, Missouri, 281-283.

[2] Ishak, K. G. and Rabin, L. (1975). Benign tumors of the liver. *Med. Clin. North Am.*, **59**, 995-1013.

[3] Nichols, F. C., Van Heerden, J. A. and Weiland, L. H. (1989). Benign liver tumors. *Surg. Clin. North Am.*, **69**, 297-314.

[4] Schumacker, H. B. (1942). Hemangioma of the liver. *Surgery*, **11**, 209-222.

[5] Nelson, R. C. and Chezmar, J. L. (1990). Diagnostic approach to hepatic hemangiomas. *Radiology*, **176**, 11-13.

[6] Quinn, S. F. and Benjamin, G. G. (1992). Hepatic cavernous hemangiomas. Simple diagnostic sign with dynamic bolous CT. *Radiology*, **182**, 545-548.

[7] Bÿrnbaum, B. A., Noz, M. E., Chapnick, J., Sanger, J. J., Megibow, A. J., Maguire, G. Q. *et al.* (1991). Hepatic hemangiomas: Diagnosis with Fusion of MR, CT, And Tc-99m- *labeled Red blood cell SPECT images Radiology*, **181**, 469-474.

[8] Adam, Y. G., Hovas, A. G. and Fortner, J. G. (1970) Gaint Hemangiomas of the liver. *Ann. Surg.*, **172**, 239-245.

[9] Belli, L., De Carlis, L., Beati, C., Rondinara, G., Sansalone, V. and Brambilla, G. (1992). Surgical treatment of symptomatic giant hemangiomas of the liver. *Surg. Gynecol. Obstet*, **174**, 474-478.

[10] Sinanan, M. and Merchiaro, T. (1989). Management of cavernous hemangioma of the liver. *Am. J. Surg.*, **157**, 519-522.

[11] Trastek, V. F., Van Heerden, J. A., Sheedy, P. F. and Adson, M. A. (1983). Cavernous hemangiomas of the liver; resect or observe? *Am. J. Surg.*, **145**, 49-53.

[12] Grieco, M. B. and Miscaff, B. G. (1978). Giant Hemangioma of the Liver. *Surg. Gynecol. Obstet.*, **147**, 783-7.

[13] Andersson, R. and Bengmark, S. (1988). Surgical Treatment of Cavernous Hemangioma of the Liver. *ACTA. Chir. Scand.*, **154**, 588-9.

[14] Kawarada, Y. and Minzumoto, R. (1984). Surgical Treatment of Giant Hemangioma of the Liver. **148**, 287-91.

[15] Berliner, L., Ferzli, G., Giantito, L. *et al.* (1983). Giant Cavernous Hemangioma of the Liver Complicated by abscess and thrombosis. *Am. J. Gastroenterol.*, **78**, 835-40.

[16] Mungovoun, J. A., Cronoun, J. J. and Vacarro, J. (1994). Hepatic Cavernous Hemangioma: Lack of Enlargement Over Time, *Radiology*, **191**, 111-13.

[17] Iwatsuki, S., Todo, S. and Starzl, T. E. (1990). Excisional therapy for benign hapatic lesions. *Surg. Gynecol. Obstet.*, **171**, 240-246.

[18] Hobbs, K. E. F. (1990). Hepatic hemangiomas. *World. J. Surg.*, **14**, 468-471.

[19] Starzl, T. E., Koep, L. J., Weil, R., Fennell, R. H., Iwatsuki, S., Kano, T. and Johnson, M. L. (1980).

Excisional treatment of cavernous hemangioma of the liver. *Ann. Surg.*, **192**, 25–27.
[20] Yamagata, M., Kanematsu, T., Matsumata, T., Utsunomiya, T., Ikeda, Y. and Sugimach, K. (1991). Management of haemangiomas of the liver: comparison of results between surgery and observation. *Br. J. Surg.*, **78**, 1223–1225.
[21] Schwartz, S. and Husser, W. C. (1987). Cavernous hemangiomas of the liver. A single institution report of 16 resections. *Ann. Surg.*, **205**, 456–463.
[22] Alper, A., Ariogul, O., Emre, A., Uras, A. and Ökten, A. (1988). Treatment of liver haemangiomas by Enucleation. *Arch. Surg.*, **123**, 660–661.
[23] Baer, H. U., Dennison, A. R., Mouton, W., Stain, S. C., Zimmermann, A. and Blumgart, L. H. (1992). Enucleation of giant hemangiomas of the liver. *Ann. Surg.*, **216**, 673–676.
[24] Petri, A., Karacsonyi, S. and Nagy, K. K. (1993). Surgical treatment of cavernous hemangiomas of the liver. *Langenbecks. Arch. Chir.*, **378**, 322–324.
[25] Kuo, P. C., Lewis, W. D. and Jenkins, R. L. (1994). Treatment of giant hemangiomas of the liver by enucleatin. *J. Am. Coll. Surg.*, **178**, 49–51.
[26] Pichlmayr, R. (1993). Chirurgische Behandlung Kavernöser hemangiome der leber. *Langenbecks. Arch. Chir.*, **378**, 319–320.
[27] Edney, J. A. (1995). Benign L_ver Tumors, *Current Surgical Therapy*, Edited by John L. Cameron, Fifth Ed. pp. 273–277 St. Louis: Mosby.

### INVITED COMMENTARY

The authors are to be congratulated for a large series of excised liver haemangiomas without mortality or serious morbidity. The paper emphasises that enucleation is not only feasible but also preferable in most instances.

Elective surgery of hepatic haemangioma is indicated if the lesion causes distressing symptoms, if the diagnosis is uncertain because of atypical appearance or concomitant malignant disease, or if it enlarges progressively.

The risk of spontaneous rupture is minimal and is not considered to justify the risk of surgical removal [1–5]. Symptoms, predominantly pain or discomfort, are related to the size and site of the lesion; they are commonly rather vague with a few exceptions like bleeding or infarction within the haemangioma (giving acute pain) or pressure on bile ducts or adjacent structures. Before surgery is comtemplated, it is therefore important to rule out other disorders that could explain the symptoms, such as gallbadder stones, peptic ulcer, reflux eosophagitis, irritable bowel syndrome. In the present series, 16/18 patients (including the ten patients who had a painful mass at physical examination) were rendered asymptomatic by surgery, which is a good outcome as compared to some other reports [4, 6]. Other authors, having a more restrictive attitude towards surgery, reported that symptoms, mainly pain, disappeared completely or became less severe with time in 21/25 patients receiving no specific treatment [4].

The authors state that formal liver resection should be performed when the haemangioma is deeply located and infiltrates the liver parenchyma. I think that the enucleation method is well suited, and the method of choice, for centrally or deeply located tumours together with blood inflow occlusion to decompress the haemangioma during removal. I do not know what the authors mean with infiltrative haemangiomas, but I agree that infiltrative lesions should be removed by a resection that gives a safe margin because of the risk of a hypervascular tumour. A confident distinction between a hypervascular tumour and a haemangioma cannot be made without tissue diagnosis. Thus, surgery should be performed if imaging studies cannot rule out malignancy or the patient has an expanding tumour or other signs of malignancy.

### *References*

[1] Trastek, V. F., van Heeden, J. A., Sheedy II, P. F. and Adson, M. A. (1983). Cavernous hemangiomas of the liver: resect or observe? *Am. J. Surg.*, **145**, 49–53.
[2] Schwartz, S. I. and Cowles Husser, W. (1987). Cavernous hemangioma of the liver: a single institution report of 16 resections. *Ann. Surg.*, **205**, 456–465.
[3] Mungovan, J. A., Cronan, J. J. and Vacarro, J. (1994). Hepatic cavernous hemangiomas – lack of enlargement over time. *Radiology*, **191**, 111–113.
[4] Farges, O., Daradkeh, S. and Bismuth, H. (1995). Cavernous hemangiomas of the liver: Are there any indications for resection? *World. J. Surg.*, **19**, 19–24.
[5] Egea, A. M., Rodriguez, R. D., Centero, M. V. and Atenza, J. A. (1996). Indications for surgery in the treatment of hepatic hemangioma. *Hepatogastroenterology*, **43**, 422–426
[6] Alper, A., Ariogul, O., Emre, A., Uras, A. and Okten, A. (1988). Treatment of liver hemangiomas by enucleation. *Arch. Surg.*, **123**, 660–661.

Karl-G Tranberg
Department of Surgery
Lund University
Lund, Sweden

